# Accelerated symptom improvement in Parkinson’s disease via remote internet-based optimization of deep brain stimulation therapy: a randomized controlled multicenter trial

**DOI:** 10.1038/s43856-025-00744-7

**Published:** 2025-01-31

**Authors:** Alireza Gharabaghi, Sergiu Groppa, Marta Navas-Garcia, Alfons Schnitzler, Laura Muñoz-Delgado, Vicky L. Marshall, Jessica Karl, Lin Zhang, Ramiro Alvarez, Mary S. Feldman, Michael J. Soileau, Lan Luo, S. Elizabeth Zauber, Benjamin L. Walter, Chengyuan Wu, Hong Lei, Damian M. Herz, Ming-Hua Chung, Yagna Pathak, Bram Blomme, Binith Cheeran, Corneliu Luca, Daniel Weiss

**Affiliations:** 1 Institute for Neuromodulation and Neurotechnology, University Hospital Tübingen (UKT), Faculty of Medicine, University Tübingen, 72076 Tübingen, Germany; 2 Center for Bionic Intelligence Tübingen Stuttgart (BITS), 72076 Tübingen, Germany; 3 German Center for Mental Health (DZPG), 72076 Tübingen, Germany; 4 Movement Disorders and Neurostimulation, Biomedical Statistics and Multimodal Signal Processing Unit, Department of Neurology, University Medical Center of the Johannes Gutenberg University, Mainz, Germany; 5 Neurosurgery Department, La Princesa University Hospital, Madrid, Spain; 6 Instituto de Investigación Sanitaria del Hospital de La Princesa, Madrid, Spain; 7 Department of Neurology, and Institute of Clinical Neuroscience and Medical Psychology, Medical Faculty and University Hospital Düsseldorf, Heinrich-Heine University Düsseldorf, Düsseldorf, Germany; 8 Unidad de Trastornos del Movimiento, Servicio de Neurología y Neurofisiología Clínica, Instituto de Biomedicina de Sevilla (IBiS), Hospital Universitario Virgen del Rocío/CSIC/Universidad de Sevilla, Sevilla, Spain; 9 Centro de Investigación Biomédica en Red sobre Enfermedades Neurodegenerativas (CIBERNED), Instituto de Salud Carlos III, Madrid, Spain; 10 Department of Neurology, Institute of Neurological Sciences, Queen Elizabeth University Hospital, Glasgow, UK; 11 Department of Neurological Sciences, Rush University, Chicago, IL USA; 12 Department of Neurology, University of California, Davis, Sacramento, CA USA; 13 Unidad de Enfermedades Neurodegenerativas, Departamento de Neurociencias, Servicio de Neurologia, Hospital Universitari Germans Trias I Pujol, Badalona, Spain; 14 Neurology, Dartmouth-Hitchcock Medical Center, Lebanon, NH USA; 15 Texas Movement Disorder Specialists, PLLC, Georgetown, TX USA; 16 Department of Neurology, Beth Israel Deaconess Medical Center, Harvard Medical School, Boston, MA USA; 17 Neurology, Indiana University School of Medicine, Indianapolis, IN USA; 18 Center for Neurological Restoration, Cleveland Clinic, Cleveland, OH USA; 19 Department of Neurological Surgery, Thomas Jefferson University, Philadelphia, PA USA; 20 Department of Neurology, University of Arizona, Tucson, AZ USA; 21 Abbott Neuromodulation, Plano, TX USA; 22 Department of Neurology, University of Miami, Miami, FL USA; 23 Center for Neurology, Department of Neurodegenerative Diseases, and Hertie Institute for Clinical Brain Research, University Tübingen, Tübingen, Germany; 24 Present Address: Feinberg School of Medicine, Northwestern University, Chicago, Illinois USA

**Keywords:** Parkinson's disease, Diseases of the nervous system, Neurological manifestations

## Abstract

**Background:**

Deep brain stimulation (DBS) has emerged as an important therapeutic intervention for neurological and neuropsychiatric disorders. After initial programming, clinicians are tasked with fine-tuning DBS parameters through repeated in-person clinic visits. We aimed to evaluate whether DBS patients achieve clinical benefit more rapidly by incorporating remote internet-based adjustment (RIBA) of stimulation parameters into the continuum of care.

**Methods:**

We conducted a randomized controlled multicenter study (ClinicalTrails.gov NCT05269862) involving patients scheduled for de novo implantation with a DBS System to treat Parkinson’s Disease. Eligibility criteria included the ability to incorporate RIBA as part of routine follow-up care. Ninety-six patients were randomly assigned in a 1:1 ratio using automated allocation, blocked into groups of 4, allocation concealed, and no stratification. After surgery and initial configuration of stimulation parameters, optimization of DBS settings occurred in the clinic alone (IC) or with additional access to RIBA. The primary outcome assessed differences in the average time to achieve a one-point improvement on the Patient Global Impression of Change score between groups. Patients, caregivers, and outcome assessors were not blinded to group assignment. Most of the data collection took place in the patient’s home environment.

**Results:**

Access to RIBA reduces the time to symptom improvement, with patients reporting 15.1 days faster clinical benefit (after 39.1 (SD 3.3) days in the RIBA group (*n* = 48) and after 54.2 (SD 3.7) days in the IC group (*n* = 48)). None of the reported adverse events are related to RIBA.

**Conclusions:**

This study demonstrates safety and efficacy of internet-based adjustment of DBS therapy, while providing clinical benefit earlier than in-clinic optimization of stimulation parameters by increasing patient access to therapy adjustment.

## Plain language summary

Deep brain stimulation (DBS) uses electrical impulses to treat disorders of the nervous system such as Parkinson’s Disease. Patients undergoing DBS need to travel to a clinic to have their treatment optimized. This study investigated whether optimizing DBS settings remotely via a mobile application leads to faster symptom improvements. The control group consisted of patients whose DBS settings were adjusted only in the clinic. Patients who had the option to adjust their therapy remotely report symptom and quality of life improvement earlier without additional side effects. These results suggest that remotely adjusting DBS settings could benefit patients and improve treatment outcomes.

## Abstract (web summary)

Gharabaghi et al assess the clinical benefit of incorporating remote internet-based adjustment (RIBA) of deep brain stimulation parameters into the continuum of care. Clinical benefits were reported more than two weeks earlier with access to RIBA, compared to in-clinic therapy optimization alone, with a similar safety profile.

## Introduction

Deep brain stimulation (DBS) has emerged as an important therapeutic intervention for several neurological and neuropsychiatric disorders, with substantial clinical benefits^1–4^. After DBS implantation, the first programming session establishes the therapeutic window for each electrode contact and preliminary therapeutic settings. Subsequent personalization of therapy (by adjusting parameters including contact configuration, pulse width, amplitude, and frequency) can take several weeks through repeated, in-person clinic visits with patients. Clinical symptoms, disease severity, electrode lead placement, and concomitant pharmacological therapy can influence adjustment of stimulation parameters. Once the stimulation parameters are optimized, maintenance visits occur once or twice a year to adjust for disease progression.

The need for frequent travel to optimize DBS poses challenges, particularly for older adults who face mobility challenges, such as walking difficulties and an increased risk of falls, as seen in patients with Parkinson’s disease (PD), and the burden on caregivers who accompany patients^5–7^. The scarcity of centers specializing in DBS for movement disorders can lead to long travel times to attend outpatient visits for DBS management. A recent study showed that patients often travel hundreds of kilometers and several hours to receive DBS treatment^8^. A survey on the availability and accessibility of DBS specialists revealed that more than one-third of patients with PD cannot easily get to a clinic due to long waiting times for an appointment or difficulty scheduling an appointment, and more than one-quarter reported difficulty contacting their clinic for advice^9^. In addition, an analysis of the Medicare claims database has shown that non-white races/ethnicities may be disproportionately affected in accessing DBS therapy^10^.

In recent decades, telemedicine has emerged as a viable healthcare option that could reduce the burden of travel and improve patients’ access to care^11^. Despite the introduction of telemedicine into healthcare since the 1990s, its integration into the DBS standard of care has been challenging. Patients with PD report access to telemedicine as a desirable, patient-centered option, and while solutions have been implemented that require the assistance of remote nurses to make physical adjustments to stimulation parameters, these involve costly trade-offs and an overall increase in the burden of care on the system^12–15^. Advances in technology allow clinicians to use telemedicine to remotely adjust DBS parameters for patients, which was first introduced and widely adopted in China during the COVID-19 pandemic^16–20^. The COVID-19 public health emergency not only disrupted clinical practice, but also clinical trials where data collection is conducted on-site at research facilities. This accelerated the adoption of study designs that use virtual tools, including telemedicine, as well as sensory technologies and wearable devices^21^.

The aim of the Remote Optimization, Adjustment and Measurement for DBS (ROAM-DBS) study was to investigate the effect of access to remote internet-based adjustment (RIBA) of stimulation parameters on DBS optimization during the early post-implantation period and to determine the practical utility of remote DBS stimulation parameter adjustment in patients with PD. The study compared two groups: patients who receive stimulation optimization after the initial on-site programming session, either in the clinic alone or with the addition of RIBA.

Patients in the RIBA group report a shorter time to achieve a patient-reported minimal clinically important improvement. Clinically important improvements in quality of life are also reported earlier in the RIBA group. At last follow-up, patient-rated and clinician-rated symptom severity and change scores improve similarly in both groups. No adverse events are reported in association with RIBA. Remote optimization of stimulation parameters can improve access to care.

## Methods

The ROAM-DBS study was a prospective, multicenter, randomized controlled trial conducted at 10 clinical sites in the United States (US) and 7 clinical sites in Europe. The study was designed as a decentralized clinical trial with most of the protocol taking place in the patients’ home environment as an ecological momentary assessment (EMA). We obtained approval of the study protocol from Beth Israel Deaconess Medical Center Institutional Review Board, Dartmouth-Hitchcock Health Institutional Review Board, Rush University Medical Center Institutional Review Board, The Cleveland Clinic Institutional Review Board, Western Institutional Review Board-Copernicus Group (covering several US sites), Comité de Ética de La Investigación con Medicamentos del Hospital Universitario de la Princesa (covering all Spanish sites), Ethik-Kommission an der Medizinische Fakultät der Heinrich Heine Universität Düsseldorf, Ethik-Kommission bei der Landesärztekammer Rheinland-Pfalz, Ethik-Kommission an der Medizinische Fakultät der Eberhard-Karls-Universität und am Universitätsklinikum Tübingen, and the North of Scotland Research Ethics Committee. The authors complied with all relevant ethical regulations when conducting the study. Patients received detailed study information and signed an informed consent form before enrolling in the study. The ROAM-DBS study is registered on clinicaltrials.gov (NCT05269862). All patients participating in ROAM-DBS are also part of the ADROIT post-market clinical follow-up study, which aims to evaluate the safety and effectiveness of DBS systems in everyday practice up to 5 years after initial programming.

### Eligibility

Investigators screened every ADROIT participant for inclusion in the ROAM-DBS study. Adult patients (≥21 years old) scheduled for de novo implantation with an Infinity DBS System with the NeuroSphere™ Virtual Clinic remote care feature (Abbott, Plano TX, USA) to treat PD were eligible for inclusion. The eligibility criteria were intended to target those patients who can use RIBA as part of their routine follow-up such as the ability of the patient or their caregiver to participate in RIBA sessions, including having appropriate internet access and the technical knowledge (or could get help from a family member or other caregiver) to operate a mobile phone. The full list of eligibility criteria can be found in Table 1.

**Table 1:** Inclusion and exclusion criteria for the ROAM-DBS study

Inclusion Criteria
The patient must be enrolled in the ADROIT clinical trial (NCT04071847)
The patient must be over 21 years old
The patient must be able to read and write
The patient must be indicated for implant with an Infinity DBS system for Parkinson’s Disease
The patient has not previously been implanted with a DBS system
The treating physician believes Virtual Clinic is appropriate as a component in the treatment regime for this patient
The patient will have access to the Virtual Clinic system through a participating site
The patient will have internet access on their Patient Controller in a location suitable for a Virtual Clinic session
The patient, or a legally acceptable representative, must provide written informed consent prior to any study-related procedure
Exclusion Criteria
The patient is currently enrolled or plans to enroll in an investigational study that may confound the results of this study
The patient has anatomic or comorbid conditions, or other medical, social, or psychological conditions that, in the investigator’s opinion, could limit the patient’s ability to participate in the study or to comply with follow-up requirements, or impact the scientific soundness of the study results.
As assessed by the treating physician, lead misplacement would prevent the DBS therapy from providing clinically meaningful benefit
The patient is unable to use the Virtual Clinic feature
The patient will not be able, in the investigator’s opinion, to demonstrate or articulate symptoms during a Virtual Clinic visit

### Baseline and randomization

Patients were enrolled no more than 6 weeks prior to planned implantation of the DBS System and at least 1 week prior to initial programming. Before implantation, all patients had a baseline visit to collect demographic information, duration of symptoms, current medications, and clinical assessments including the Movement Disorders Society Unified Parkinson’s Disease Rating Scale (MDS-UPDRS) and Parkinson’s Disease Questionnaire-39 (PDQ-39)^22,23^. All but three of the randomized patients received an iPhone™ for decentralized collection of Patient Reported Outcomes (PROs) and an Apple Watch™ to test the feasibility of behavioral and physiological monitoring including longitudinal, remote monitoring of motor symptoms in PD. During the start of the study, there was a delay in obtaining the home monitoring kits for 3 patients.

**Fig. 1:**
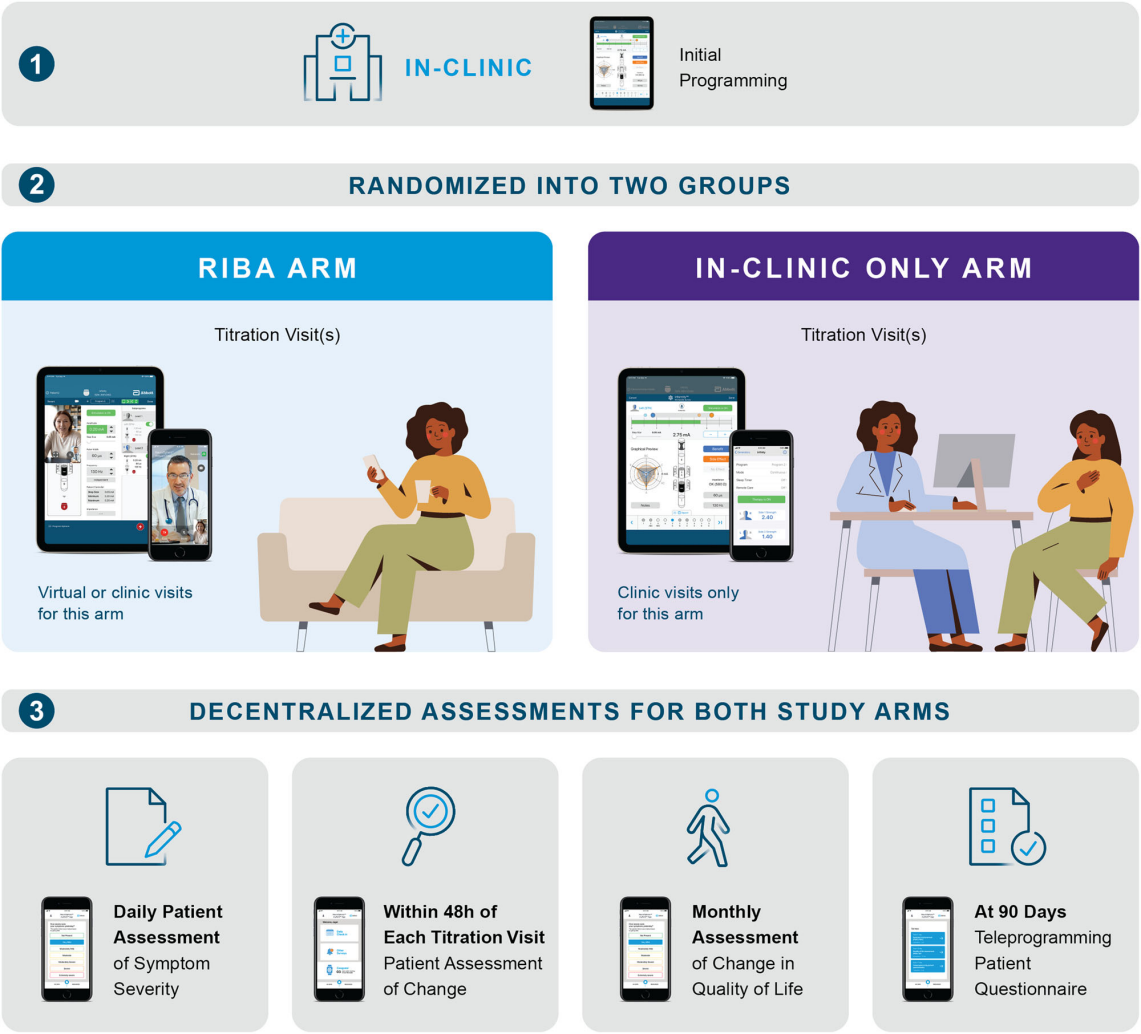
Overview of the decentralized data collection. A decentralized data collection process took place in both study arms. Initial programming occurred in the clinic and consisted of a monopolar review in which the therapeutic window was established for each electrode contact. Subsequent titration visits occurred in-clinic and/or remotely via Virtual Clinic (RIBA arm) or in-clinic only. All patient assessments, the daily assessment of symptom severity (PGI-S), the assessment of symptom change (PGI-C) within 48 hours of the titration visit, and the monthly assessment of change in Quality of Life (PDQ-39), could be performed via a mobile application designed specifically for this study. Clinician assessment of symptom change (CGI-C) could only be performed via an electronic data recording system.

Patients diagnosed with PD undergoing DBS were randomly assigned in a 1:1 ratio according to a predefined randomization scheme, blocked into groups of 4, concealed allocation, and no stratification. An independent statistician was responsible for creating the automated randomization schedule and this was blinded from the statistical team conducting the analyses. Assignment of patients to a cohort was not based on characteristics of the patient, or the judgement of the enrolling site personnel. The randomization scheme allocated patients on a first-come, first-served basis, so sites were not guaranteed to receive consecutive randomization entries for their patients. This, in addition to the blocking schedule, prevented the prediction of randomization assignment. After surgery and initial on-site stimulation parameter configuration, the intervention involved adjustment of DBS settings either in-clinic only (IC) or through additional access to the RIBA program. Randomization after the initial programming ensured that there was no bias in the initial programming based on the expected treatment group. No blinding was performed after randomization and patients, caregivers, and clinicians were aware of the assigned treatment arm. All procedures in this study were in accordance with the standard of care of each participating center and the medical necessity for each patient. The design of the investigational PRO applications on the mobile device enabled push notifications timed to the endpoints of the study, allowing for high, on-time response rates. Figure 1 provides an overview of the decentralized data collection process.

### Primary Endpoint

Assessment of symptom changes relative to those experienced after initial programming (Patient Global Impression of Change; PGI-C) was collected following a titration session. PGI-C is a 7-point scale depicting a patient’s rating of overall improvement. Patients rate their change as 1: very much improved, 2: much improved, 3: minimally improved, 4: no change, 5: minimally worse, 6: much worse, or 7: very much worse. Patients were asked to complete the PGI-C survey within 48 hours of the titration session. The predefined primary endpoint entailed assessing the duration until symptom amelioration, quantified by a minimal clinically important difference (MCID) of one-point improvement (minimally improved) on the PGI-C^24^. An intention-to-treat (ITT) analysis for the primary endpoint included all randomized patients. A per-protocol (PP) analysis included patients who received the treatment (i.e., stimulation optimization format) as randomized. Patients in the RIBA group who did not receive a remote titration session were excluded from PP analysis. Patients in the IC group who received a RIBA session were excluded from PP analysis.

The ROAM-DBS study had about 90% power to detect a 2-week (14 days) time difference between two arms to achieve a one-point improvement in PGI-C with α = 0.05 when at least 48 patients participated in the follow-up of 90 days. This was based on a two-sample t-test that allowed for unequal variance at a two-sided significance level of 5%. We assumed that one additional titration session would be required to achieve a one-point improvement in PGI-C score after initial programming. In addition, the mean time between initial programming and the time at which the patient achieved a one-point improvement on the PGI-C scale was estimated as four weeks (28 days) with a standard deviation of four weeks (28 days) for in-clinic only sessions, and one week (7 days) with a standard deviation of one week (7 days) for the group who had access to RIBA. Under these assumptions, the expected effect size Cohen’s *d* between the study groups to achieve a one-point improvement on the PGI-C scale was calculated to be 1.03. Finally, we assumed a 5% attrition rate at three months in both cohorts due to withdrawal, missed visit, or lost to follow-up.

### Secondary Endpoints and Additional Analyses

The analyses for secondary endpoints were based on the PP population. Secondary endpoints included the daily global assessment of symptom severity (Patient Global Impression of Severity; PGI-S). The PGI-S asked the following: “How severe were your symptoms yesterday?” with response options of 1: not present, 2: very mild, 3: mild, 4: moderate, 5: moderate severe, 6: severe, and 7: extremely severe. The safety endpoint of the ROAM-DBS trial was the rate of stimulation optimization-related serious adverse events (SAEs) in the two study arms and was not powered due to the low expected rate of adverse events (AEs). AEs were considered serious if they resulted in death, chronic disease, hospitalization, life-threatening illness or injury, permanent impairment to a body structure or function, or a medical or surgical intervention to prevent these. Three independent qualified clinicians adjudicated safety events. Clinicians assessed changes in global impression of the patient’s symptom severity and improvement relative to the initial programming (Clinician Global Impression of Severity; CGI-S and Clinician Global Impression of Change; CGI-C) after each titration session. Scoring was done in the same way as for PGI-S and PGI-C.

Patients completed the PDQ-39 assessment of their quality of life monthly. While the PGI and CGI are one-dimensional assessments of symptoms, the PDQ-39 provides a multidimensional assessment of the patient’s quality of life, providing additional information about the impact of therapy beyond changes in symptoms. The 39 items of the questionnaire represent eight domains (mobility, activities of daily living, emotional well-being, stigma, social support, cognition, communication, and bodily discomfort) consisting of 3–10 items each.

At 90 days, patients in the RIBA group completed a custom 5-point Likert survey (1: Strongly agree, 2: Agree, 3: Neither agree nor disagree, 4: Disagree, 5: Strongly disagree) that included the questions: “the Virtual Clinic system was easy to use” and “I would use the Virtual Clinic system again”. Patients with in-clinic stimulation parameter adjustment after initial programming were asked: “About how long (in hours and minutes) did it take you to have your DBS system programmed in person? This may include travel to and from the clinic, parking, time in the clinic etc.” and “About how far do you travel (in miles or km) to have your DBS system programmed in person?”. Patients in the RIBA group were asked: “About how long (in hours and minutes) did it take you to have your DBS system programmed using Virtual Clinic session? This may include setting up your Patient Controller or other preparation activities.” Finally, after each titration conducted remotely, clinicians were asked: “Was the Virtual Clinic session an adequate replacement for in-clinic programming? (Were you able to complete the necessary steps for analysis and programming?)”. Response options were Yes or No.

The data were uploaded to a cloud-based secure server supporting RIBA. In cases where this was not feasible, forms were manually collected by the clinic during titration visits. Upon completion of the survey, iPhones and Apple watches were returned to the study sites. The IC group was offered access to RIBA after the 12-week study period.

To calculate the duration of RIBA sessions, information was extracted from the Virtual Clinic backend sever^25^. Sessions were excluded from analysis if they were considered a trial session (duration less than 5 min), or if the patient instead of the clinician ended the session. The latter carries the risk that not all program updates are saved, resulting in an incomplete session. Session durations were summed for the day, to include sessions where a reconnection occurred after a disconnected session (e.g., poor connection), or sessions where the clinician was able to successfully end the session and reconnect later to confirm that the new settings are acceptable.

### Statistical analysis

Statistical analysis was completed using SAS version 9.4 (SAS Institute Inc., Cary, NC, USA). The powered primary endpoint is calculated at α = 0.05 level to test the difference in Restricted Mean Survival Time (RMST) between RIBA and IC groups. RMST is typically defined as the average event-free survival time up to a pre-specified time point, which is calculated as the area under the Kaplan-Meier survival curve. RMST in this study refers to the average time of achieving at least one-point improvement in PGI-C up to 90 days. Categorical data are presented as proportions and continuous variables are summarized with means and standard deviations (SD). 95% confidence intervals (CIs) are provided to compare intervention arms for non-powered endpoints. Line graphs were developed using GraphPad Prism (GraphPad Software, La Jolla, CA, USA).

## Results

### Study population

**Fig. 2:**
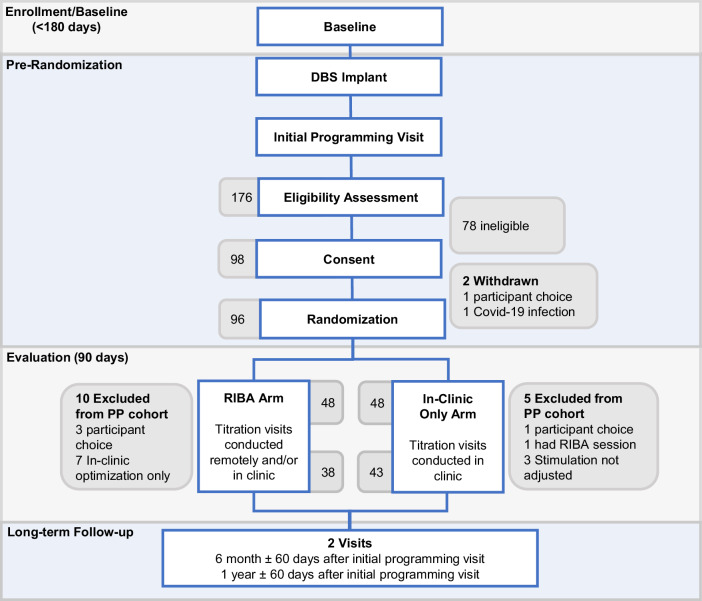
CONSORT Flow Diagram. For the two study arms, the number of patients excluded from the per-protocol (PP) analysis is shown.

Between March 2022 and July 2023, 176 patients at 18 centers in the US and Europe were screened for eligibility. The most common reasons for ineligibility were lack of internet for a RIBA session and the patient’s inability to use the remote care application. A total of 98 participants were enrolled, of whom 48 were allocated to adjust stimulation parameters in the clinic only, and 48 to receive additional RIBA. Two patients were not randomized; one patient felt uncomfortable wearing the digital watch, handling the mobile phone, and potentially getting randomized in the RIBA arm and the other patient was infected with COVID-19, which delayed DBS surgery for too long to be included in the study. The overall dropout rate was 17% (17 participants), with 43 patients in the IC arm and 38 patients in the RIBA arm completing follow-up at Day 90 (Fig. 2). Demographics and baseline scores of the RIBA and IC groups are presented in Table 2. In both groups, more than 90% of patients received bilateral DBS of the subthalamic nucleus (STN). Two patients in the RIBA group were stimulated unilaterally in the STN and one patient was stimulated bilaterally in the internal segment of the globus pallidus (GPi). One patient in the IC group was stimulated unilaterally in the STN and two patients were stimulated bilaterally in the GPi.

**Table 2:** Demographics and baseline assessments of the per-protocol population

	RIBA Arm, N=38	IC Arm, N=43
Percent Male (Sex Assigned at Birth)	65.8% (25/38) 95% CI: 48.6% to 80.4%	74.4% (32/43) 95% CI: 58.8% to 86.5%
Age at consent (years)	62.4 (SD 9.9) 95% CI: 59.1 to 65.7	62.0 (SD 7.6) 95% CI: 59.6 to 64.3
Symptom Duration (years)	11.0 (SD 4.0) 95% CI: 9.7 to 12.3	10.6 (SD 5.3) 95% CI: 9.0 to 12.3
Time since Diagnosis (years)	9.1 (SD 3.4) 95% CI: 8.0 to 10.2	8.4 (SD 4.2) 95% CI: 7.1 to 9.7
Height (cm)	170.8 (SD 7.3) 95% CI: 168.4 to 173.2	174.3 (SD 8.7) 95% CI: 171.6 to 177.0
Weight (kg)	78.4 (SD 16.4) 95% CI: 73.0 to 83.8	79.2 (SD 15.4) 95% CI: 74.4 to 83.9
Baseline PDQ-39 Summary Index	29.9 (SD 13.6) 95% CI: 25.4 to 34.3	23.4 (SD 12.8) 95% CI: 19.3 to 27.5
Baseline MDS-UPDRS part III OFF Med/OFF Stim	46.8 (SD 15.8) 95% CI: 41.5 to 52.2	43.3 (SD 16.9) 95% CI: 37.6 to 49.0
Baseline MDS-UPDRS part III ON Med/OFF Stim	23.3 (SD 10.2) 95% CI: 19.8 to 26.8	21.8 (SD 11.0) 95% CI: 18.3 to 25.3
Baseline MDS-UPDRS II	16.3 (SD 6.9) 95% CI: 14.1 to 18.6	13.7 (SD 6.7) 95% CI: 11.5 to 15.8
Baseline MDS-UPDRS IV	8.2 (SD 4.9) 95% CI: 6.6 to 9.9	7.8 (SD 4.7) 95% CI: 6.3 to 9.3
**Type of Parkinson’s Disease**		
Tremor dominant	26.3% (10/38) 95% CI: 13.4% to 43.1%	53.5% (23/43) 95% CI: 37.7% to 68.8%
Akinetic-rigid	34.2% (13/38) 95% CI: 19.6% to 51.4%	23.3% (10/43) 95% CI: 11.8% to 38.6%
Mixed symptoms	39.5% (15/38) 95% CI: 24.0% to 56.6%	23.3% (10/43) 95% CI: 11.8% to 38.6%

Results are presented as proportion or mean (standard deviation) with 95% confidence intervals.

### Primary endpoint and associated analyses

**Fig. 3:**
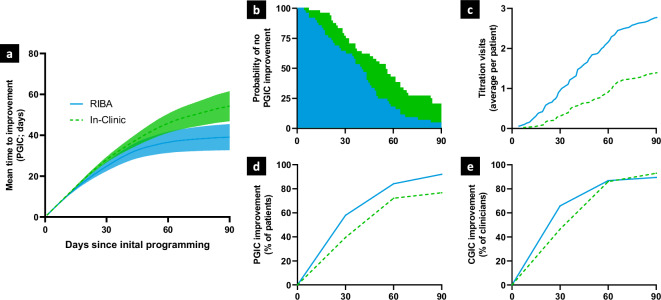
Primary endpoint and associated analyses. All panels show the same x-axis of days since initial programming and use equivalent colors for Remote Internet-Based Adjustment (RIBA) (blue line) and In-Clinic (IC) (green dashed line) groups. **a** Mean time to at least one point improvement on the Patient Global Impression of Change (PGI-C), the minimal clinically important difference (MCID) on this scale, over the 90-day study period in the intention-to-treat (ITT) cohort. Colored area around the lines represents 95% confidence interval. **b** Probability of not achieving at least one-point PGI-C improvement over the 90-day study period in both study arms. Area under the Kaplan-Meier survival curve represents the mean time to at least one-point improvement on the PGI-C up to 90 days. Panels a and b contain data from *n* = 48 independent individuals in both study groups (ITT cohort) **c**) mean number of cumulative titration visits per patient over the 90-day study period in the RIBA and IC groups. **d**, **e** Percentage of patients whose symptoms improved by at least one-point on the Global Impression of Change Scale as rated by the patient (PGI-C) and clinician (CGI-C) over the 90-day study period in both study arms, respectively. Panels **c**, **d**, and **e** contain data from *n* = 38 independent individuals in the RIBA group and *n* = 43 independent individuals in the IC group (Per-Protocol cohort).

An ITT analysis of the primary endpoint revealed that patients who were optimized in the RIBA group reported at least one point of improvement on the PGI-C at 39.1 (SD 3.3) days after initial programming over a 90-day follow-up period. In contrast, patients who were optimized in the IC group reported at least one point of improvement at 54.2 (SD 3.7) days after initial programming indicating a clear positive effect of access to RIBA on mean time to improvement (difference of 15.1 days between groups; two-sided RMST test for equality with significance Level=0.05, *p* = 0.0022) (Fig. 3a, b). The PP analysis equally showed strong evidence for accelerated clinical benefit via RIBA access, reporting 15.9 days faster MCID improvement in the RIBA group (after 36.8 (SD 3.7) days in the RIBA group and after 52.7 (SD 3.9) days in the IC group; two-sided RMST test for equality with significance Level=0.05, *p* = 0.0033). Cumulatively at 90 days, four patients in the RIBA group and six patients in the IC group reported clinically relevant worsening of symptoms (i.e., one-point worsening on the PGI-C).

At 30 and 60 days, 58% (22/38) [95% CI: 40.8%, 73.7%] and 84% (32/38) [95% CI: 68.7%, 94.0%] of patients in the RIBA group and 40% (17/43) [95% CI: 25.0%, 55.6%] and 72% (31/43) [95% CI: 56.3%, 84.7%] of patients in the IC group reported at least one point improvement on the PGI-C. At 90 days, 92% (35/38) [95% CI: 78.6% to 98.3%] and 77% (33/43) [95% CI: 61.4% to 88.2%] of patients cumulatively reported at least minimal clinical benefit in the RIBA and IC groups, respectively (Fig. 3d). At 30 days, 66% (25/38) [95% CI: 46.0%, 78.2%] and 47% (20/43) [95% CI: 31.2%, 62.3%] of clinicians reported at least one point improvement on the CGI-C. The clinician’s assessment on the CGI scale showed more similarity between groups at 60 and 90 days with 87% (33/38) [95% CI: 71.9%, 95.6%] and 90% (34/38) [95% CI: 75.2%, 97.1%] of clinicians in the RIBA group and 86% (37/43) [95% CI: 72.1%, 94.7%] and 93% (40/43) [95% CI: 80.9% to 98.5%] of clinicians in the IC group cumulatively reporting at least minimal clinical benefit (Fig. 3e).

The RIBA arm received an average of 2.8 (95% CI: 2.2 to 3.4) titration visits after initial programming, while the IC arm received 1.5 (95% CI: 1.3 to 1.7) titration visits (Fig. 3c). In the RIBA arm, patients participated in an average of 2.2 (95% CI: 1.8 to 2.7) RIBA sessions and 0.6 (95% CI: 0.4 to 0.8) in-clinic sessions. Half of the patients in the RIBA arm (19/38) received RIBA sessions only.

### Global impression scales at 90 days

The PGI-C score was almost identical, 2.4 (SD 0.7) vs. 2.6 (SD 1.7) (mean difference: −0.2; 95% CI: −1.4 to 1.0) for RIBA and IC groups, respectively. A similar result was obtained when clinicians assessed clinical benefit (CGI-C) 90 days after initial programming, 2.1 (SD 1.0) vs. 1.8 (SD 0.8) (mean difference: 0.4; 95% CI: −0.5 to 1.2). Both study arms had comparable outcomes at 90 days on the PGI severity scale (PGI-S), with baseline scores of approximately 5 points, 5.1 (SD 1.0) vs. 5.1 (SD 0.9), corresponding to moderately severe, which improved to approximately 3 points, 3.1 (SD 1.2) vs. 2.8 (SD 1.1), corresponding to mild (mean difference: 0.3; 95% CI: -0.3 to 0.8) in RIBA and IC groups, respectively. Clinicians reported similar improvement in disease severity (CGI-S) in both groups, with baseline scores of 5.5 (SD 0.8) vs. 5.3 (SD 0.9), moderately severe to severe, improving to 2.9 (SD 1.3) vs. 3.1 (SD 1.0), mild (mean difference: −0.2; 95% CI: −1.2 to 0.9).

### Quality of Life Assessment

**Fig. 4:**
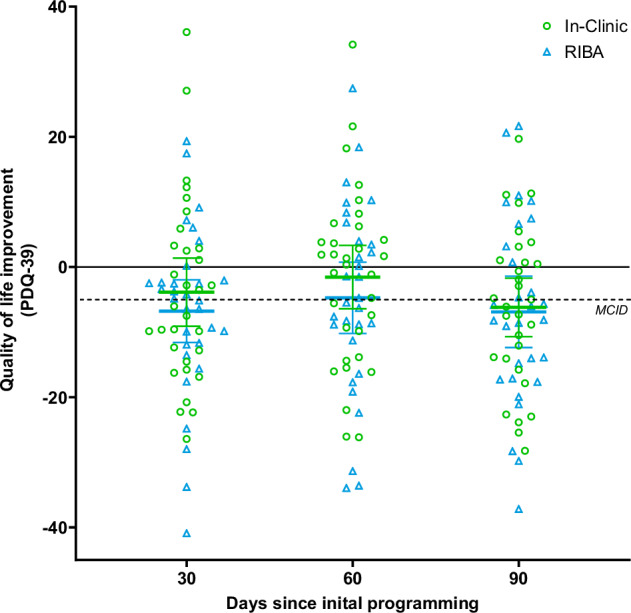
Improvement in Parkinson’s Disease Questionnaire-39 (PDQ-39) summary index relative to baseline up to 90 days since initial programming in both study arms. Individual data points from the two groups are presented at 30, 60, and 90 days. Blue triangles represent the results for the Remote Internet-Based Adjustment (RIBA) group and green circles represent the results for the In-Clinic (IC) group. The dashed black line indicates the minimal clinically important difference (MCID) of 5-point improvement (reduction). The horizontal lines represent the mean values of the RIBA (blue) and IC (green) groups. Error bars represent 95% confidence interval. This figure contains data from *n* = 31, *n* = 30, and *n* = 29 independent participants in the RIBA group and *n* = 31, *n* = 32, and *n* = 30 independent participants in the IC group at 30, 60, and 90 days, respectively.

Figure 4 shows the changes in the PDQ-39 summary index (SI) from pre-implant baseline to 30, 60, and 90 days after initial programming. Both groups showed an improvement (reduction) in PDQ-39 SI of 6 to 7 points at 90 days. Similar to ratings on global impression scales, this improvement was reported earlier, after 1 month, and sustained until 3 months in the RIBA group, whereas in the IC group a similar mean MCID improvement (5-point reduction from baseline) was reported at 3 months only. Point estimates in both study groups and between-group differences for the eight PDQ-39 domains at 30, 60, and 90 days are shown in Supplementary Table 1. Changes from baseline in the two study groups and between-groups differences in change from baseline for the eight PDQ-39 domains at 30, 60, and 90 days are shown in Table 3.

**Table 3:** PDQ-39 Quality of Life domains changes between baseline and 30, 60, and 90 days, respectively, in the RIBA and IC groups and mean change difference (95% confidence interval) between the two study arms at these timepoints

	Timepoint	Activities of Daily Living	Bodily Discomfort	Cognition	Communication	Emotional Well-being	Mobility	Social Support	Stigma
RIBA Mean ± SD (n) [95% CI]	30 Days	−15.0 ± 23.5 (32) [−23.5, −6.5]	−7.6 ± 24.3 (32) [−16.3, 1.2]	−0.6 ± 18.8 (31) [−7.5, 6.3]	−5.2 ± 15.9 (32) [−11.0, 0.5]	−4.7 ± 15.5 (32) [−10.3, 0.9]	−10.0 ± 21.0 (32) [−17.6, −2.4]	1.8 ± 16.3 (32) [−4.1, 7.7]	−11.3 ± 25.1 (32) [−20.4, −2.3]
IC Mean ± SD (n) [95% CI]		−11.4 ± 24.2 (31) [−20.3, −2.6]	−5.6 ± 26.2 (31) [−15.3, 4.0]	−2.6 ± 17.3 (31) [−9.0, 3.7]	−2.7 ± 21.1 (31) [−10.4, 5.1]	−4.3 ± 15.0 (31) [−9.8, 1.2]	−6.6 ± 24.7 (31) [−15.7, 2.4]	5.2 ± 18.1 (31) [−1.4, 11.9]	−2.8 ± 22.2 (31) [−11.0, 5.3]
Difference [95% CI]		3.5 [−8.5,15.6]	1.9 [−10.8, 14.6]	−2.0 [−11.2, 7.2]	2.5 [−6.9, 12.0]	0.4 [−7.3, 8.1]	3.4 [−8.2, 15.0]	3.4 [−5.3, 12.1]	8.5 [−3.4, 20.4]
RIBA Mean ± SD (n) [95% CI]	60 Days	−14.6 ± 24.4 (31) [−23.6, −5.7]	−4.0 ± 27.9 (31) [−14.3, 6.2]	1.0 ± 16.4 (31) [−5.0, 7.0]	−3.5 ± 20.7 (31) [−11.1, 4.1]	0.3 ± 19.2 (31) [−6.8, 7.3]	−9.8 ± 25.1 (30) [−19.2, −0.4]	1.3 ± 18.6 (31) [−5.5, 8.2]	−9.3 ± 24.7 (31) [−18.3, −0.2]
IC Mean ± SD (n) [95% CI]		−10.4 ± 23.4 (32) [−18.8, −2.0]	−5.2 ± 25.8 (32) [−14.5, 4.1]	1.4 ± 16.2 (32) [−4.5, 7.2]	0.8 ± 16.8 (32) [−5.3, 6.9]	0.4 ± 15.2 (32) [−5.1, 5.9]	−2.1 ± 30.0 (32) [−12.9, 8.7]	8.7 ± 16.7 (32) [2.7, 14.8]	−5.9 ± 16.3 (32) [−11.7, 0.0]
Difference [95% CI]		4.2 [−7.8, 16.3]	−1.2 [−14.7, 12.4]	0.4 [−7.9, 8.6]	4.3 [−5.3, 13.8]	0.1 [−8.6, 8.9]	7.7 [−6.3, 21.8]	7.4 [−1.6, 16.3]	3.4 [−7.2, 14.0]
RIBA Mean ± SD (n) [95% CI]	90 Days	−16.4 ± 25.2 (30) [−25.8, −7.0]	−6.5 ± 22.4 (31) [−14.7, 1.8]	−2.4 ± 18.2 (31) [−9.1, 4.3]	−8.3 ± 21.5 (31) [−16.2, −0.4]	−3.2 ± 22.4 (31) [−11.4, 5.0]	−6.2 ± 21.2 (30) [−14.1, 1.7]	0.9 ± 22.6 (31) [−7.3, 9.2]	−11.1 ± 20.5 (31) [−18.6, −3.6]
IC Mean ± SD (n) [95% CI]		−14.6 ± 24.8 (34) [−23.2, −5.9]	−6.2 ± 26.4 (32) [−15.8, 3.3]	−1.8 ± 18.7 (34) [−8.4, 4.7]	−2.9 ± 16.7 (34) [−8.8, 2.9]	−3.2 ± 15.8 (33) [−8.8, 2.5]	−8.1 ± 23.6 (31) [−16.8, 0.5]	2.8 ± 15.1 (34) [−2.5, 8.1]	−11.9 ± 16.4 (33) [−17.8, −6.1]
Difference [95% CI]		1.8 [-10.7, 14.3]	0.2 [-12.1, 12.5]	0.6 [-8.6, 9.7]	5.4 [-4.2, 15.0]	0.1 [-9.7, 9.8]	-2.0 [-13.5, 9.5]	1.9 [−7.8, 11.5]	-0.8 [-10.2, 8.5]

### Burden and acceptance of RIBA program

After 90 days of DBS therapy, patients from both arms completed a custom survey about the burden of stimulation parameter adjustment and their acceptance of the RIBA program. Patients in the IC group reported traveling an average of 102.5 (SD 158.0) km to attend titration sessions at the clinic. Consequently, the time required to optimize the DBS system (including preparation, travel, and any overnight stays) in person was considerably longer, as patients in the IC group reported 15.2 (SD 39.3) hours to complete the adjustment of stimulation parameters in the clinic. This contrasted with a mean time of 0.8 (SD 1.3) hours to complete stimulation optimization through RIBA, including preparatory activities such as patient controller setup. Regarding the acceptance of RIBA, when asked to rate the statement “I would use the virtual clinic system again”, the mean response score was 1.7 (SD 0.7), which came closest to the answer ‘Agree’. A total of 96% of patients responded Strongly Agree or Agree to this question. Similarly, most patients agreed that the virtual clinic system was easy to use; mean response score was 2.0 (SD 0.8). A total of 84% of patients responded Strongly Agree or Agree to this question. Finally, clinicians indicated that remote stimulation parameter adjustment was an adequate substitute for in-clinic stimulation optimization after 88% (44/50) of RIBA sessions, because they were able to complete the necessary steps for analysis and stimulation parameter adjustment. This question was asked after each titration visit conducted remotely and the data were analyzed for clinicians who adjusted stimulation parameters in-clinic and remotely in the study to avoid bias in the responses.

### Duration of RIBA sessions

The mean duration of RIBA sessions in this study was 14 minutes and 05 seconds (SD 08 minutes and 03 seconds), and the median duration was 11 minutes and 37 seconds (range 05 minutes and 01 seconds and 40 minutes and 44 seconds), for a total of 78 titration sessions.

### Adverse events

The number of AEs reported was consistent with the expected rate for DBS (Table 4). In the RIBA group, four SAEs were reported in three patients: a lead misplacement requiring surgical revision, an additional surgery to repair a loose suture in the scalp, followed by an infection of the implanted components that required complete removal of the DBS system a week later, and a patient who developed a subdural hematoma after a fall. Similarly, five SAEs were reported in three patients in the IC group. One patient had an infection requiring Implantable Pulse Generator (IPG) replacement, developed acute psychosis, and had reported cardiac dysfunction for which a pacemaker was implanted. The latter event was considered unrelated to the procedure or device. One patient fell shortly after increasing the stimulation amplitude using the patient controller and was taken to the emergency department where he reported feeling dizzy and lightheaded. A CT scan of the brain was unremarkable for cerebral hemorrhage or acute intracranial pathology. One patient was admitted to the hospital with fever symptoms; this patient was not part of the PP cohort because he was not optimized during the study. No AE, serious or non-serious, was reported in association with RIBA. In the IC group, two non-serious AEs were reported in one patient. This patient reported increased confusion, short-term memory loss, and blurred vision for four days after DBS surgery.

**Table 4:** Rate of adverse events

AE Type	Sub-grouping	RIBA Arm	IC Arm
		N events/ N patients	N events/ N patients
Non-Serious	All DBS related AE	0/0	2/1
	Device, or Procedure related AE	0/0	2/1
	Stimulation related AE	0/0	0/0
Serious	All DBS related SAE	4/3	5/3
	Device, or Procedure related SAE	4/3	4/2
	Stimulation related SAE	0/0	1/1

Serious adverse events (SAE) are separated from nonserious events (AE).

## Discussion

This is the first prospective randomized controlled trial to assess remote DBS stimulation optimization, and the first decentralized DBS study based on EMAs. Patients with PD treated with DBS achieved MCID in the primary patient-reported outcome more than two weeks earlier when therapy optimization was done through RIBA compared with IC adjustment of stimulation parameters. Both groups had patients who reported no additional improvement after initial programming, although the proportion of patients reporting clinical benefit on PGI-C at 90 days was higher in the RIBA group (92% vs 77%). These findings suggest that the integration of RIBA into the standard of care program for DBS patients reduces the time needed to optimize therapy by facilitating access to care.

Specifically, the RIBA arm received an average of 1.3 additional titration visits after initial programming. Initial programming sessions averaged approximately 1 hour but lasted up to 4 hours in some patients, consistent with reports in literature^26,27^. In our clinical experience, follow-up titration sessions in the clinic last twenty minutes to one hour appointment blocks. RIBA sessions in this study lasted an average of 14 minutes. This short duration indicates that, despite a higher number of titration visits, the total patient-clinician interaction time will be comparable to or even less than IC stimulation optimization. Furthermore, an assessment of the total number of titration visits (and associated use of healthcare resource) cannot be made until at least one year after the initial programming. It is conceivable that patients may experience clinical benefit earlier due to improved access to care, but the number of interactions (including maintenance visits) may ultimately be comparable to conventional in-clinic adjustment of stimulation parameters. The additional travel burden associated with in-clinic stimulation optimization was also assessed. Patients (and caregivers) reported traveling an average of 103 km (64 mi) and 15 hours to prepare, travel, and attend titration sessions at the clinic. As with the distance traveled, the total time was highly variable, with the 95% CI ranging from 2.5 to 28 hours. Patients who indicated that titration in the clinic took 24 hours or more required an overnight hotel stay due to long-distance travel or were patients who required at least one night in the hospital for observation, at the discretion of the investigator. These findings are consistent with previous observations that patients and clinicians experience fewer barriers to scheduling and attending sessions with remote stimulation parameter adjustment, as reflected in both the short duration of RIBA sessions and the patient-reported time burden associated with remote versus in-person visits^8,28^.

At 30 days after implantation, the percentage of patients and clinicians reporting an improvement of at least one point on the global impression scale was numerically higher in the RIBA group than in the IC group. At this timepoint, the percentage difference between the two groups was even greater in the clinician’s assessment of improvement, reaffirming that RIBA leads to faster optimization in DBS patients. This difference remained at 60 and 90 days for patient assessment of improvement but was no longer the case for clinician assessment of improvement at these later timepoints. Previous research across disease states has shown relatively poor agreement between patient and clinician responses when assessing quality of life and disease severity, although to our knowledge no data have been collected for PD or other neurological disorders^29–31^. The clinician’s evaluation of improvement should be considered complementary to the patient’s evaluation.

Two clinically important observations provide additional context to these results. First, although any observed differences between the groups are due to chance, there was a higher proportion of PD patients with a tremor-dominant phenotype in the IC group. Motor symptom scores improve more in tremor-dominant PD patients than an akinetic-rigid phenotype^32,33^. In-clinic and remotely, tremor is a consistent symptom (compared to bradykinesia or gait) that responds within seconds of a parameter change^34^. Second, the three patients receiving unilateral STN stimulation differed from the overall rate of improvement on PGI-C, with one patient experiencing MCID improvement at the end of the 90-day period (in the IC group) and one patient not improving within 90 days after implantation (in the RIBA group). The three patients who received bilateral GPi stimulation reported time to at least minimal clinical benefit consistent with the overall rate. More patients with these types of stimulation are needed to substantiate these results, as our study is representative of patients receiving bilateral STN stimulation.

In addition to the effects observed on the global impression scales, DBS was associated with a substantial improvement in quality of life, as evidenced by a mean PDQ-39 improvement of approximately 6 to 7 points at 90 days in both groups, which surpasses the clinically relevant change on this scale of 5-points^35^. Consistent with other assessments, patients in the RIBA group reported this improvement in quality of life earlier. Analysis of individual PDQ-39 domains revealed no differences in improvement from baseline between groups, although a trend was noted for the communication domain, which improved more than 5 points more in the RIBA group at 90 days; in line with expected responses to improved access to care for these patients.

The rate of adverse events related to DBS was low in both groups. One serious adverse event related to stimulation or stimulation parameter optimization occurred in the IC group; one patient fell after stimulation adjustment. These findings suggest that RIBA does not carry a risk beyond that of DBS therapy. These results are consistent with the known safety profile of DBS and confirm that serious adverse events in this cohort are generally related to the implantation procedure rather than to stimulation or optimization effects. This is an important finding as there is relatively little experience with remote DBS stimulation parameter adjustment, and concerns about emerging patient risks may influence whether RIBA is used. In addition, significant efforts have been made to protect RIBA sessions and patient data. The remote care platform is configured with multiple layers of security and meets internationally recognized security standards. Full technical details of the security architecture of the remote care platform have been reported previously^25^.

This study, conducted in patients with advanced PD, was designed as an EMA, a type of research that uses applications on smart devices to send notifications at prespecified intervals to prompt patients to answer questions or questionnaires. EMAs study the thoughts and behavior of patients in their daily lives by repeatedly collecting data in their normal environment and are often more accurate than data collection methods that require patient recall^36^. EMA and telemedicine applications could also be useful in the assessment and treatment of a range of psychiatric disorders. For example, EMA has led to new insights into the temporal patterns of obsessive-compulsive disorder (OCD) symptoms^37^. In addition, OCD and some forms of depression are associated with agoraphobia, where patients experience anxiety when leaving familiar surroundings or using public transport^38,39^. Digital health tools that can be accessed from home may be an important first step toward better treatment.

Li et al. (2017) compared remote stimulation optimization of DBS with sham stimulation in patients with PD and found remote adjustment of stimulation parameters to be effective, but the study lacked a comparison with an active control of stimulation according to standard in-clinic optimization^40^. A retrospective study by Chen and colleagues (2022) compared in-clinic stimulation parameter adjustment, remote adjustment, and a combination of in-clinic and remote adjustment, and found similar results in all arms^41^. Because this study was conducted retrospectively, patients and clinicians were able to choose the most attractive optimization option, which could potentially introduce a bias that limits the generalizability of the results. To our knowledge, our prospective, randomized controlled trial is the first to report level 1 clinical data on the effects of remote adjustment of stimulation parameters in a PD patient population treated with DBS.

Remote monitoring of PD patients is another telemedicine application that has gained interest in recent years. This usually involves smartphone applications that enable remote motor symptoms and cognitive assessments. Two feasibility studies have reported generally high levels of compliance with smart device applications for remote monitoring among PD patients^42,43^. This is consistent with the current study in which we achieved 80% compliance for the PGI-C assessment within 48 hours of the titration visit (and greater than 90% compliance when the 48-hour restriction is lifted) and the monthly PDQ-39 assessment. Patients completed on average two-thirds of the daily symptom severity assessment. Non-compliance in Motolese et al.‘s study was generally related to loss of interest, psychological distress, absence of a caregiver, the presence of falls, and a higher level of clinical disability^42^. In both studies, clinicians were able to correlate the remotely collected data with clinical outcomes and individual PD profiles, suggesting their usefulness in daily practice and in combination with other digital health tools such as remote optimization of stimulation, although studies with larger sample size and for longer periods are warranted.

Temporary reimbursement policies were in place for some regions due to the COVID-19 pandemic. The Centers for Medicaid & Medicaid Services (CMS) has classified remote DBS stimulation optimization as a category 3 service that is likely to have a clinical benefit when delivered via telehealth, but there is not yet sufficient evidence to justify permanent coverage (https://www.cms.gov/newsroom/fact-sheets/final-policy-payment-and-quality-provisions-changes-medicare-physician-fee-schedule-calendar-year-1). According to CMS, an example of clinical benefit includes a more rapid beneficial treatment response, which is consistent with the results we present in this study. Barriers to adopting remote care still exist in several countries, including digital literacy, acceptance, and cost^44^. Furthermore, in the US, insurance can require a clinician to be licensed to practice medicine in the state where the patient resides, which can be a deterrent to the use of telemedicine. The results presented will hopefully motivate supranational unions, federal states, and individual regions to implement telemedicine-friendly policies, for the benefit of these vulnerable patient populations.

Further work is needed to investigate long-term outcomes beyond the 90 days evaluated in this study. The ROAM-DBS patients will continue in the ADROIT study, which will follow patients for up to 5 years, allowing for long-term evaluation of outcomes and safety with remote optimization of stimulation parameters. In addition, future additional data may complement the findings presented here, including objective digital data from wearables collected during the ROAM-DBS study period. Finally, research is underway to investigate the relationship between factors such as distance to the clinic, access to the internet, patient technical skills, and PROs.

There are limitations worth noting. First, the patients and providers were not blinded. This allowed the study to capture how patients and clinicians adjusted the frequency of adjustments in response to randomization. Although it is possible that patients or clinicians were more highly motivated when they were randomized to the RIBA arm, patients reported on average an additional 14 hours to attend the stimulation optimization in the clinic, compared to RIBA sessions performed at home, suggesting that other factors are responsible for the results presented. Second, PD patients undergoing DBS are typically highly motivated and represent a younger, less cognitively impaired PD population. A requirement for inclusion was that the patient had to be able to work with the remote care application (or have a caregiver to support) and have access to a stable and reliable internet connection. These factors limit the generalizability of the results to telemedicine applications within the overall PD population. In contrast, a strength of this study was the inclusion of a substantial number of patients from 17 sites in different geographical locations. Third, while this study evaluated the time patients spent attending the titration sessions, comparable clinician time was not collected. A recent study evaluated over 40 million specialty visits and, of the 24 specialties evaluated, 16 had more in-person follow-up after an office visit than after a telehealth visit, including pain medicine, which uses similar hardware and stimulation optimization^45^. Further work is needed to determine how the incorporation of remote adjustment of stimulation parameters might affect hospital workflows.

In conclusion, this randomized controlled trial demonstrated that the clinical benefits of DBS were seen earlier in patients receiving internet-based adjustments compared to the current standard of care in-clinic stimulation optimization, with a comparable safety profile. Patients in the RIBA group reported good acceptance of remote adjustment of stimulation parameters and had easier access to their treating clinician, while the total time required for optimization was comparable to in-clinic visits due to the short duration of the sessions. Without the need to travel, the time burden associated with DBS stimulation optimization is considerably reduced. This remotely accessible, scalable digital care intervention could lead to a paradigm shift in DBS care by providing access to care at home and helping to overcome treatment barriers.

### Inclusion and ethics

The investigators participating in this study were all experts in movement disorders, balancing gender and geographic region as much as possible. The ROAM-DBS study addressed two of the commonly cited critical barriers to inclusion: logistical resources (time, caretaking responsibilities, and transportation limitations), and a lack of infrastructure in underserved areas. The RIBA program and PROs used in this study were direct ways to increase diversity by eliminating the need to come to the clinic to participate in clinical research. The ROAM-DBS study also enabled deeper penetration into traditionally underserved populations, such as rural or economically disadvantaged communities that may lack traditional clinical research infrastructure. However, internet access is less common in low-income households, especially among older people in poverty, which remained a barrier for more inclusion and diverse patient populations (https://aspe.hhs.gov/sites/default/files/private/pdf/263601/Internet_Access_Among_Low_Income.pdf). With the further deployment of Edge, 3 G, 4 G, and 5 G networks worldwide, we expect that cellular connections stable (and affordable) enough to enable RIBA will only increase in the future. The results presented can help increase access to care and reduce barriers to inclusion in the real world.

## Supplementary information

Supplementary Table

Description of Additional Supplementary Files

Supplementary Data 1

Supplementary Data 2

## Data Availability

In more detail to the statement on clinicaltrials.gov: de-identified individual participant data collected during the ROAM-DBS trial will be shared at 2 years after article publication with no end date. These data will be available to researchers who provide a methodologically sound proposal for the purposes of achieving specific aims outlined in that proposal. Proposals should be directed to Devyani Nanduri via email devyani.nanduri@abbott.com and will be reviewed by the ROAM-DBS study management committee. Requests to access data to undertake hypothesis-driven research will not be unreasonably withheld. To gain access, data requesters will need to sign a data access agreement and to confirm that data will only be used for the agreed purpose for which access was granted. The source data for Fig. 3 is in Supplementary Data 1 and the source data for Fig. 4 is in Supplementary Data 2.
