# Supplementary material for: Accelerated symptom improvement in Parkinson’s disease via remote internet-based optimization of deep brain stimulation therapy: a randomized controlled multicenter trial
Source: Commun Med (Lond). 2025 Jan 31;5:31. doi: 10.1038/s43856-025-00744-7 (PMC11785990; doi:10.1038/s43856-025-00744-7)
Supplement: Supplementary file 1 — Supplementary Table [file 43856_2025_744_MOESM1_ESM.pdf]

Supplementary Table 1. PDQ-39 Quality of Life domains at baseline, and at 30, 60, and 90 days in the RIBA and IC groups and mean differences (95% confidence interval) between the two study arms at these timepoints.

	Timepoint	Activities of Daily Living	Bodily Discomfort	Cognition	Communication	Emotional Well-being	Mobility	Social Support	Stigma
RIBA Mean (n) SD	Baseline	39.1 (38) SD 25.0	35.5 (38) SD 22.3	24.0 (38) SD 16.5	24.3 (38) SD 18.4	30.3 (38) SD 21.6	42.8 (38) SD 23.3	16.1 (38) SD 20.8	26.6 (38) SD 24.3
IC Mean (n) SD		31.5 (40) SD 23.0	29.3 (39) SD 22.3	20.9 (40) SD 18.0	19.4 (40) SD 15.7	23.0 (40) SD 17.4	33.1 (40) SD 23.2	9.7 (40) SD 14.8	20.8 (40) SD 18.3
Difference [95% CI]		-7.7 [-18.5, 3.2]	-6.3 [-16.4, 3.9]	-3.1 [-10.9, 4.7]	-5.0 [-12.7, 2.8]	-7.2 [-16.1, 1.6]	-9.6 [-20.1, 0.9]	-6.4 [-14.6, 1.8]	-5.9 [-15.6, 3.9]
RIBA Mean (n) SD	30 Days	25.7 (32) SD 19.1	27.9 (32) SD 21.9	25.0 (31) SD 15.1	19.3 (32) SD 18.3	24.5 (32) SD 20.2	33.6 (32) SD 21.1	18.7 (32) SD 20.1	14.5 (32) SD 16.3
IC Mean (n) SD		23.5 (34) SD 19.6	28.4 (34) SD 20.3	19.7 (34) SD 14.9	18.1 (34) SD 21.2	21.3 (34) SD 18.6	30.9 (34) SD 24.0	5.2 (34) SD 18.1	19.5 (34) SD 22.4
Difference [95% CI]		-2.1 [-11.6, 7.4]	0.6 [-9.8, 11.0]	-5.3 [-12.8, 2.1]	-1.1 [-10.8, 8.6]	-3.2 [-12.7, 6.4]	-2.7 [-13.8, 8.4]	-1.3 [-10.3, 7.6]	5.0 [-4.6, 14.6]
RIBA Mean (n) SD	60 Days	26.5 (31) SD 20.5	30.4 (31) SD 24.0	23.4 (31) SD 14.0	19.6 (31) SD 20.0	27.2 (31) SD 22.8	32.1 (30) SD 24.9	17.5 (31) SD 15.7	17.5 (31) SD 20.8
IC Mean (n) SD		23.7 (34) SD 18.4	27.2 (34) SD 20.1	21.5 (34) SD 16.4	21.3 (34) SD 20.7	25.5 (34) SD 21.7	33.0 (34) SD 25.1	8.7 (34) SD 16.7	16.0 (34) SD 14.2
Difference [95% CI]		-2.8 [-12.5, 6.9]	-3.2 [-14.2, 7.9]	-1.9 [-9.4, 5.6]	1.7 [-8.4, 11.8]	-1.7 [-12.7, 9.4]	0.9 [-11.6, 13.4]	1.6 [-6.7, 10.0]	-1.5 [-10.5, 7.4]
RIBA Mean (n) SD	90 Days	22.9 (30) SD 17.6	28.0 (31) SD 24.7	22.4 (31) SD 15.2	17.2 (31) SD 17.9	24.2 (31) SD 19.9	34.7 (30) SD 21.0	18.5 (31) SD 17.4	13.3 (31) SD 13.4
IC Mean (n) SD		18.4 (36) SD 15.7	24.3 (35) SD 18.3	17.9 (37) SD 16.2	19.4 (37) SD 18.4	19.1 (36) SD 19.2	25.8 (34) SD 21.2	12.8 (37) SD 14.1	9.7 (36) SD 13.1
Difference [95% CI]		-4.5 [-12.8, 3.8]	-3.7 [-14.5, 7.2]	-4.5 [-12.1, 3.1]	2.2 [-6.7, 11.0]	5.1 [-14.7, 4.5]	-8.9 [-19.4, 1.7]	-5.7 [-13.5, 2.1]	-3.6 [-10.1, 2.9]
