# Supplementary material for: Accelerated symptom improvement in Parkinson’s disease via remote internet-based optimization of deep brain stimulation therapy: a randomized controlled multicenter trial
Source: Commun Med (Lond). 2025 Jan 31;5:31. doi: 10.1038/s43856-025-00744-7 (PMC11785990; doi:10.1038/s43856-025-00744-7)
Supplement: Supplementary file 2 — Description of Additional Supplementary Files [file 43856_2025_744_MOESM2_ESM.pdf]

Description of Additional Supplementary Files

File name: Supplementary Data 1

File description: The source data for Figure 3

File name: Supplementary Data 2

File description: The source data for Figure 4
